# Neural Decoding Reveals Concurrent Phonemic and Subphonemic Representations of Speech Across Tasks

**DOI:** 10.1162/nol_a_00034

**Published:** 2021-05-07

**Authors:** Sara D. Beach, Ola Ozernov-Palchik, Sidney C. May, Tracy M. Centanni, John D. E. Gabrieli, Dimitrios Pantazis

**Affiliations:** 1 McGovern Institute for Brain Research, Massachusetts Institute of Technology, Cambridge, MA, USA; 2 Program in Speech and Hearing Bioscience and Technology, Harvard University, Cambridge, MA, USA; 3 Lynch School of Education and Human Development, Boston College, Chestnut Hill, MA, USA; 4 Department of Psychology, Texas Christian University, Fort Worth, TX, USA

**Keywords:** speech perception, categorical perception, neural decoding, multivariate pattern analysis, MEG

## Abstract

Robust and efficient speech perception relies on the interpretation of acoustically variable phoneme realizations, yet prior neuroimaging studies are inconclusive regarding the degree to which subphonemic detail is maintained over time as categorical representations arise. It is also unknown whether this depends on the demands of the listening task. We addressed these questions by using neural decoding to quantify the (dis)similarity of brain response patterns evoked during two different tasks. We recorded magnetoencephalography (MEG) as adult participants heard isolated, randomized tokens from a /ba/-/da/ speech continuum. In the passive task, their attention was diverted. In the active task, they categorized each token as *ba* or *da*. We found that linear classifiers successfully decoded *ba* vs. *da* perception from the MEG data. Data from the left hemisphere were sufficient to decode the percept early in the trial, while the right hemisphere was necessary but not sufficient for decoding at later time points. We also decoded stimulus representations and found that they were maintained longer in the active task than in the passive task; however, these representations did not pattern more like discrete phonemes when an active categorical response was required. Instead, in both tasks, early phonemic patterns gave way to a representation of stimulus ambiguity that coincided in time with reliable percept decoding. Our results suggest that the categorization process does not require the loss of subphonemic detail, and that the neural representation of isolated speech sounds includes concurrent phonemic and subphonemic information.

## INTRODUCTION

Speech perception is defined as “the process that transforms speech input into a phonological representation of that input” (Samuel, 2011, p. 50). Whether that process is attributed to categorization (Holt & Lotto, 2010), pre-lexical abstraction (Obleser & Eisner, 2009), or recovery of the speaker’s motor intention (Liberman & Mattingly, 1985), the brain must undoubtedly solve a many-to-one mapping problem when confronted with a world of highly acoustically variable phoneme realizations. One clue to the nature of the neural solution to this problem is the behavioral phenomenon of categorical perception, in which sounds that vary continuously along acoustic dimensions are perceived to fall into discrete, linguistically meaningful categories, which suggests that the neural representation of speech input may undergo a rapid—perhaps obligatory—loss of subphonemic detail. In this study, we explored whether time-resolved multivariate analyses and techniques for capturing representational structure would reveal such a transformation. We report a novel application of these approaches to studying the categorical perception of isolated speech syllables and the fate of subphonemic detail under different task demands.

### Categorical vs. Continuous Perception: Behavior

Evidence of categorical speech perception comes from laboratory paradigms in which participants perform identification and discrimination tasks on stimuli drawn from a synthetic acoustic continuum. Perception is said to be categorical when discrimination performance is predicted by the identification function: Two stimuli identified as belonging to different categories will be well discriminated, while two equally distant stimuli belonging to the same category will be poorly discriminated. This pattern is observed reliably for consonants more so than for vowels (Altmann et al., 2014; Eimas, 1963; Pisoni, 1973), both for voicing continua (e.g., /da/-/ta/, /ba/-/pa/) and for place-of-articulation continua (e.g., /ba/-/da/, /da/-/ga/). Nevertheless, sensitivity to within-category differences has been demonstrated experimentally (Barclay, 1972; Massaro & Cohen, 1983; Pisoni & Tash, 1974; Samuel, 1977), suggesting that listeners can and do access subphonemic detail under some conditions. Indeed, positing the existence of both an auditory (continuous) mode and a phonemic (categorical) mode of perception has long been an empirically supported compromise position (Dehaene-Lambertz et al., 2005; Pisoni, 1973).

### Categorical vs. Continuous Perception: Neuroimaging

Evidence from functional magnetic resonance imaging (fMRI) suggests that specific cortical regions, principally in the left hemisphere, perform categorical processing of speech input above and beyond acoustic analysis: the superior temporal sulcus (Joanisse et al., 2007; Liebenthal et al., 2005), the supramarginal gyrus (Joanisse et al., 2007; Raizada & Poldrack, 2007), and the inferior frontal sulcus (Myers et al., 2009). The diversity of results across studies may be due to differences in the task performed in the scanner (e.g., passive habituation, active discrimination, monitoring for an orthogonal stimulus dimension), and to the way that neurocognitive processes of various latencies and durations manifest in low time-resolution fMRI.

Methods with superior time resolution have also been used to test the intuition that speech processing proceeds through a series of stages and transformations from acoustic details to phoneme representations and beyond. As early as ∼50 ms after stimulus onset, auditory evoked fields carry information about a consonant’s place of articulation (Gage et al., 2002; Obleser et al., 2003; Tavabi et al., 2007). However, results at ∼100 ms have not been unequivocal. An event-related potentials (ERP) study demonstrated continuous encoding of voice onset time in N1 amplitude (Toscano et al., 2010), while an electrocorticography study reported that categorical place-of-articulation information was strongest at 110–150 ms (E. F. Chang et al., 2010). A magnetoencephalography (MEG) study characterized the N100m as “higher-level and abstract” (Tavabi et al., 2007, p. 3161), while an ERP study found that the P300 still reflected “subtle phonetic changes” (J. Y. Lee et al., 2019, p. 129). If there is indeed a transformation from continuous encoding of acoustic-phonetic detail to abstract categories, it has not been definitively identified. Moreover, it is not clear from prior reports whether the emergence of the abstract category involves the loss of acoustic-phonetic detail. As with the fMRI studies reviewed above, the top-down influences of various task demands may have contributed to the differences observed among studies.

### Task Effects

Since the initial report of categorical speech perception (Liberman et al., 1957), further research has shown that its canonical behavioral patterns respond to manipulations of task structure and stimulus context. For example, categorical responses to speech-like formant patterns depend on perceiving the stimuli as speech and not as another type of environmental sound (Mattingly et al., 1971); prior experience with sequential presentation and discrimination of continuum items makes perception less categorical (Pisoni & Lazarus, 1974); selective exposure to one endpoint of a continuum shifts the location of listeners’ category boundary (Diehl, 1975). In other words, the (dis)similarity of items along a continuum is malleable. However, the effect on perception of asking listeners to categorize or label stimuli cannot be measured from behavior because passive listening, as a baseline condition, has no direct output.

To understand the effects of task, researchers have turned to neuroimaging and neural recording while manipulating behavioral relevance, often by directing attention to one stimulus attribute to the exclusion of others, or by contrasting the presence vs. absence of goal-directed behavior. Selective attention exerts measurable influence on the neural representations that underpin perceptually guided behaviors, a phenomenon that has been extensively documented in the visual domain (Bugatus et al., 2017; Cukur et al., 2013; Erez & Duncan, 2015; Nastase et al., 2017), as well as in speech perception (e.g., attending to one of two speakers [Mesgarani & Chang, 2012] or attending to the speaker vs. the content [Bonte et al., 2014]). More relevantly to the present study, the presence vs. absence of an explicit task (i.e., directing attention to phonology) during speech presentation engages more left-lateralized brain networks (reviewed in Turkeltaub & Coslett, 2010), but the effect on neural *representations* of speech is far from clear.

A handful of studies have directly compared neural representations evoked by passive exposure to speech sounds with those evoked during either an incidental listening task or active categorization of the stimuli, but with mixed results. Feng et al. (2018) identified abstract representations of Mandarin lexical tones in fMRI that generalized across passive and active tasks, although this study did not address how an acoustic continuum gives way to a categorical tone representation. Bidelman and Walker (2017) found that ERPs elicited by a vowel continuum patterned categorically into *prototypical* and *ambiguous*, but only during active categorization. Using MEG, Alho et al. (2016) observed phonetic category-specific adaptation to a /da/-/ga/ continuum during an incidental listening task but not during passive exposure. However, at the same early latencies of this adaptation, Chang et al. (2010) demonstrated categorical neural response patterns with only passive exposure to a /ba/-/da/-/ga/ continuum. This raises the possibility that a categorical transformation is an obligatory part of bottom-up speech processing, such as might be undertaken by a specialized biological module (Liberman & Mattingly, 1985). Probing this unresolved question was a motivation of the present experiment.

### A Neural Decoding Approach

Two gaps in the categorical perception literature can be uniquely addressed with a neural decoding approach. First, it is unknown whether categorical perception is the result of a bottom-up/obligatory or a top-down/task-driven process. We address this question by varying task demands during MEG: The task’s effect on stimulus dissimilarity is indexed not by behavioral discrimination, but by the extent to which the evoked neural patterns are correctly classified by a machine-learning algorithm. Second, it is unknown whether stimulus-classification accuracy will reveal that subphonemic detail decays vs. persists over time, and how this phenomenon might be modulated by task demands. Previous studies of continuum perception were able to infer category structure based on whether a stimulus change of a given magnitude yielded release from adaptation (e.g., Altmann et al., 2014) or a mismatch response (e.g., Dehaene-Lambertz, 1997; Sharma & Dorman, 1999), but these paradigms may influence the strength and timing of decodable information by establishing perceptual expectations (Demarchi et al., 2019; Kok et al., 2017). Here, we perform a direct comparison of active vs. passive task demands on the representation of isolated, randomized speech tokens by examining neural pattern dissimilarity with high temporal resolution, inferring category structure from the classifier’s ability to discriminate pairs of stimuli.

Broadly, we expected that active vs. passive listening conditions would affect the readout of neural information. One hypothesis was that the demands of the active task would non-selectively boost the decoding of stimuli via attentional mechanisms. Attention and behavioral relevance are known to strengthen stimulus representations such that they can be reliably decoded from neural patterns (Bonte et al., 2014; Erez & Duncan, 2015; Kaiser et al., 2016). Relatedly, because the active task would require a perceptual decision (and, eventually, a motor response), we hypothesized that representations would be maintained (i.e., decodable) for a longer time in active trials than in passive trials—a correlate of working memory and/or decision processes (S. H. Lee & Baker, 2016). Time-resolved neural decoding analyses have previously distinguished different stages of information processing that contribute to categorization and decision-making (De Lucia et al., 2012).

Another hypothesis was that the active task would reshape the neural representation of an acoustic continuum to reflect the nonlinear way it is perceived, with less pattern dissimilarity within categories and greater pattern dissimilarity between categories, supporting the cognitive demand of category assignment. Such a result has been obtained with neural recordings in the auditory cortices of mice that had learned to categorize high- and low-frequency sounds; importantly, this reorganization of tuning occurred during the active task but not during passive listening (Xin et al., 2019). To our knowledge, no study in humans has examined how a categorization task affects the structure of neural representations of auditory continuum stimuli. However, there may be a parallel in the visual domain: Like the acoustic-phonetic properties of speech, color varies along a continuum whose subtle gradations are discriminable with effort, but humans divide up the space into just a few discrete categories. In an intriguing fMRI experiment that compared a color-naming task to diverted attention, the task not only strengthened the signal in visual areas, but also induced categorical clustering of neural color spaces, aligned with participants’ perception of categories along the color spectrum (Brouwer & Heeger, 2013). Thus, we hypothesized that a phoneme-naming task might alter the structure of neural representations in a similar top-down manner. The correlate of this hypothesis is that subphonemic detail would be discarded as categories emerged, much as in the conscious experience of categorical perception, in which only the abstract representation rises to the level of awareness. A neural decoding approach allows us to determine whether information about stimulus goodness or ambiguity nevertheless persists in neural patterns.

Finally, MEG offers the opportunity to examine hemispheric lateralization with excellent temporal resolution. Unlike the electroencephalography (EEG) signal, magnetic fields are not distorted as they pass through the head. Importantly, the superior-temporal location of auditory cortex yields magnetic dipoles that are oriented tangential to the scalp with topographies that straightforwardly distinguish auditory-evoked activity in each hemisphere (Gutschalk, 2019). Using multivariate decoding, we expected to replicate previous findings of categorical phoneme processing in the left hemisphere and explored whether the right hemisphere perhaps contained reliable but low-intensity patterns of information that were not captured by prior univariate neuroimaging analyses.

### The Present Study

In this study, we recorded MEG while adult participants encountered the same 10 continuum stimuli under passive listening conditions (diverted attention) and active listening conditions (overt stimulus labeling as *ba* or *da*). In the first part of the analysis, we performed a binary classification of the labels assigned to stimuli in the active task in order to confirm that the MEG data contain sufficient information to decode the two phoneme percepts. We compared the results obtained from performing classification on all sensors, left-hemisphere sensors only, and right-hemisphere sensors only. The time-resolved decoding time series should reveal the emergence and dissipation of this perceptual information during the trial.

In the second part of the analysis, we measured overall neural pattern dissimilarity in order to ask whether task demands affect the strength and/or maintenance of neural stimulus representations. Here we conducted pairwise decoding of all 45 possible stimulus pairings at each time point in the trial’s processing cascade. If the active task increases the strength of representations, we should observe significantly higher overall decoding accuracy at a given time; if the active task increases the maintenance of representations, we should observe significantly higher overall decoding accuracy over a period of time.

In the final part of the analysis, we explored whether task demands affect the structure of neural stimulus representations—that is, whether subphonemic detail is lost over time and/or differentially in the two tasks. We took three approaches to assaying the structure of continuum representation. First, we employed the traditional categorical-perception analysis of comparing the pairwise neural dissimilarity of equidistant stimuli falling within vs. across the listener-defined category boundary. Second, we used representational similarity analysis (RSA; Kriegeskorte et al., 2008), correlating pairwise neural dissimilarity matrices with models of stimulus perception and stimulus ambiguity. Third, we performed *k*-means clustering on the neural dissimilarities. If the loss of subphonemic detail is an obligatory part of bottom-up speech processing, we should observe that similar structure emerges in active and passive tasks: namely, low within-category dissimilarity, high between-category dissimilarity, high correlations with the perceptual model, low correlations with the ambiguity model, and distinct phonemic clusters.

## MATERIALS AND METHODS

### Participants

Twenty-four right-handed adults (13 male/11 female; mean age = 26 years, *SD* = 6 years) participated. All were native speakers of American English. To confirm self-reports of normal hearing, pure-tone detection thresholds were measured in the left and right ears at standard audiometric frequencies (250, 500, 1000, 2000, 4000, and 8000 Hz); all thresholds were ≤30 dB. Each participant provided written, informed consent prior to the experiment, whose procedures were approved and overseen by the Committee on the Use of Humans as Experimental Subjects at the Massachusetts Institute of Technology.

### MEG Tasks

#### Stimuli

The auditory stimuli for the two MEG tasks were 10 syllables constituting an acoustic continuum. The original 20-step continuum was constructed by Stephens and Holt (2011) via linear predictive coding between natural-speech /ba/ and /da/ tokens uttered by an adult male speaker of American English. For this experiment, we used the 10 odd-numbered steps of the original continuum and renumbered them from 1 (/ba/) to 10 (/da/). Syllables were 310 ms in duration. Stimuli were delivered via insert earphones (Etymotic, Oak Grove Village, IL) at a comfortable listening level, consistent across participants.

#### Passive task

**Figure 1.**
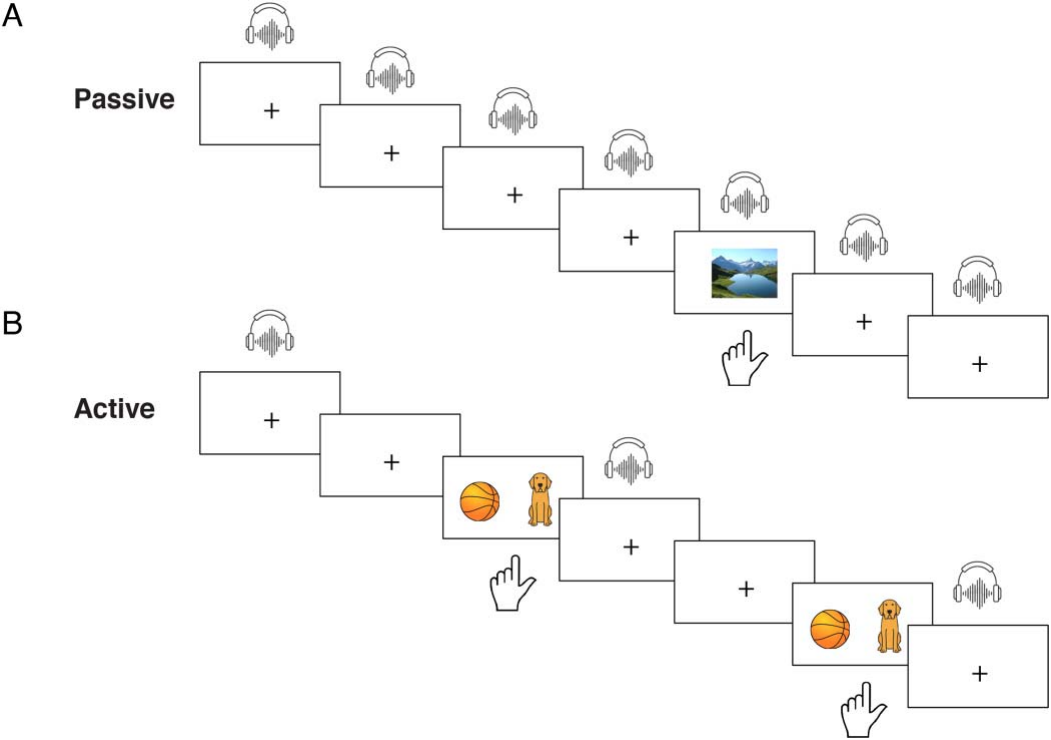
Task design. (A) Passive task. Participants were passively exposed to isolated, randomized tokens from the 10-step /ba/-/da/ speech continuum while their attention was diverted to a visual detection task. One auditory stimulus was presented on each trial; participants pressed a button when a photograph appeared. (B) Active task. Participants were exposed to the same stimuli, but were asked to label each token as either *ba* or *da* via button press. Counterbalanced response options appeared after a delay period (see text for details); *ball* signified *ba* and *dog* signified *da*.

Participants were passively exposed to 43 tokens each of the 10-step continuum in pseudorandom order. As a cover task, and to maintain arousal, they were instructed to maintain visual fixation and to press a button with the left index finger each time a photograph appeared on the screen. They were told that they would hear sounds in their earphones but that they could ignore them. Each trial consisted of the presentation of one auditory syllable, with the inter-trial interval jittered randomly among 1,410, 1,610, and 1,810 ms (measured from sound onset). The fixation point (a white plus sign on a black background) was maintained throughout the experiment, except on target trials, on which a photograph (a nature scene) appeared synchronously with the auditory stimulus (Figure 1A). Task duration was approximately 12 min. Button-presses and response times (with respect to stimulus onset) were recorded for behavioral analysis, but the 30 target trials were discarded from MEG analysis, yielding a total of 400 passive trials, 40 per stimulus. No motor responses were required on the trials included in the neural analyses. The Passive task was performed immediately before the Active task.

#### Active task

Participants were asked to label each of 40 tokens of each of the 10 continuum steps, presented in pseudorandom order, as either *ba* or *da*. On each trial, an auditory syllable was presented, and after a delay of 900 ms measured from sound onset, two response options appeared on the screen: a cartoon ball (representing *ba*) and a cartoon dog (representing *da*) (Figure 1B). (As these data were collected as part of a larger cross-sectional investigation including participants without orthographic expertise, we defined the categories not with the letters *b* and *d*, but in terms of the initial sounds in *ball* and *dog*.) Participants were instructed to press the button under their left middle finger to select the option on the left side of the screen and the button under their left index finger to select the option on the right side of the screen. During the first half of the task, one response option always appeared on the left and the other always appeared on the right. At the midpoint of the task, an experimenter spoke to the participant via intercom and reminded them that each response option would now appear on the other side of the screen. Which response option appeared first on the left vs. the right was counterbalanced across participants. An inter-trial interval of random duration between 250 and 750 ms was initiated by button-press. In order to prevent contamination of the MEG data of interest with preparatory motor activity, button-presses were not accepted until the response options appeared, and participants were asked to delay their motor responses until this time. Response times were not analyzed for the Active task because no instructions regarding speed or accuracy were given, and because participants were able to use the button press to advance the experiment at their preferred pace. Task duration was approximately 15 min, and this task also yielded 400 active trials, 40 per stimulus.

### MEG Acquisition and Preprocessing

MEG was recorded from each participant during the Passive and Active tasks on an Elekta Triux 306-channel system (102 magnetometers and 204 planar gradiometers) with a sampling rate of 1000 Hz and online filtering between 0.03 and 330 Hz. Continuous head position measurements were recorded from five coils affixed to the scalp. Prior to recording, three anatomical landmarks (nasion, left and right preauricular points) were registered with respect to the head-position coils using a Polhemus digitizer. Raw data were preprocessed with Maxfilter software (Elekta, Stockholm), incorporating head-movement correction and spatiotemporal filtering of noise sources originating from outside the MEG helmet. Subsequent processing was conducted in Brainstorm (Tadel et al., 2011). Eye-blink and cardiac artifacts were removed from the continuous dataset using signal-space projection. Trials were epoched from −200 to 1,000 ms with respect to the onset of the auditory stimulus, baseline-corrected with respect to the prestimulus period, and low-pass filtered at 15 Hz. Additionally, data from each sensor were *z*-normalized for the subsequent multivariate analysis using the baseline mean and standard deviation. Feature normalization prevents distance-based classification by linear support vector machines (see next section) from being dominated by features with larger scales at the expense of features with smaller scales, which is a risk when combining data from magnetometer and planar-gradiometer sensor types.

### Multivariate Pattern Analysis

We used multivariate pattern analysis (MVPA) to derive measures of neural dissimilarity for (a) stimuli presented during the Passive task; (b) stimuli presented during the Active task; and (c) stimuli presented during the Active task that were subsequently labeled *ba* vs. *da*, regardless of stimulus identity. For (a) and (b)—hereafter referred to as *stimulus decoding*—the output was a 10 × 10 symmetric neural representational dissimilarity matrix (RDM) for each participant and time point and task, in which each cell contained the decoding accuracy of the row stimulus vs. the column stimulus. For (c)—hereafter referred to as *percept decoding* or *ba vs. da decoding*—the output was a single decoding-accuracy time course for each participant.

**Figure 2.**
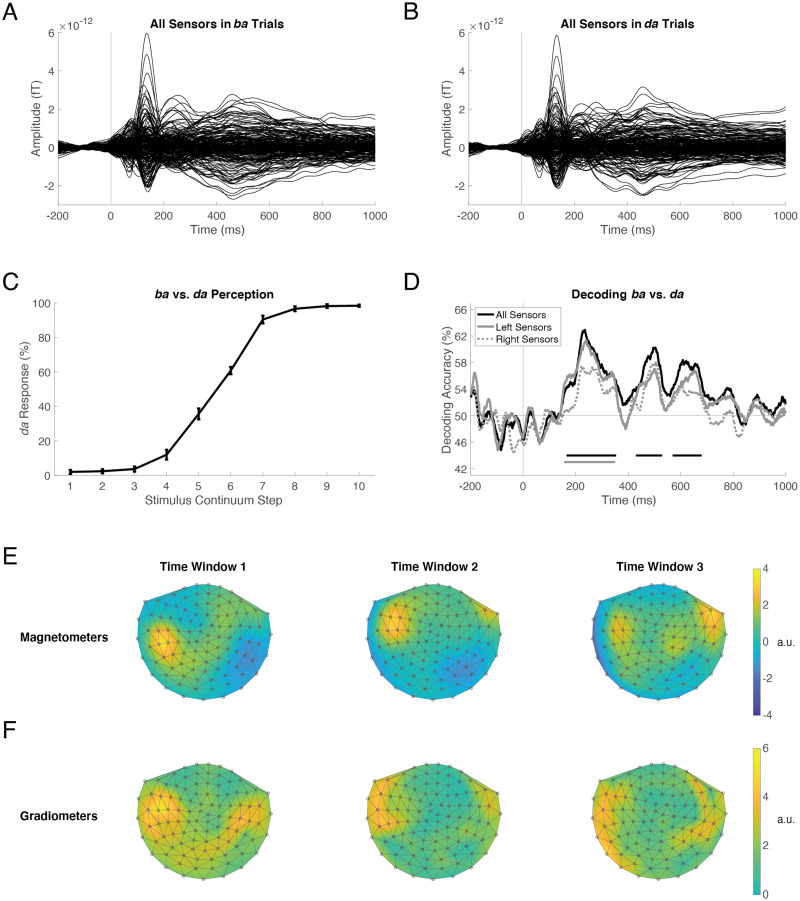
Decoding *ba* vs. *da* perception in the Active task. MEG sensor amplitudes averaged over trials and participants, for all trials perceived as *ba* (A) and all trials perceived as *da* (B). Each trace is one of 306 sensors, which together formed the multivariate pattern for training and testing the “all sensors” classifier at each time point (1-ms resolution). Decoding was also performed using left-hemisphere and right-hemisphere sensor subsets. Stimulus onset is at 0 ms. (C) Participants’ behavioral responses during the Active task demonstrate categorical perception of the /ba/-/da/ continuum, with substantial ambiguity at steps 5 and 6. Error bars represent the standard error of the mean. (D) The time course of *ba* vs. *da* decoding, averaged across participants, performed in all sensors (black), left-hemisphere sensors (solid gray), and right-hemisphere sensors (dotted gray). Horizontal lines below the traces in corresponding colors indicate time windows of significantly above-chance decoding accuracy. (E, F) Topographical plots of the transformed classifier weights averaged within the first, second, and third time windows during which all-sensors decoding was significantly above chance. Magnetometers (E) and planar gradiometers (F) are plotted separately. Left is left and top is anterior. The values in each plot have been divided by the standard deviation to yield arbitrary units (a.u.).

MVPA was performed using linear support vector machines (SVM) as implemented in LIBSVM 3.21 (C.-C. Chang & Lin, 2011) for MATLAB (MathWorks, Natick, MA). SVM classification was performed for each participant separately and at each time point (1-ms resolution) independently, with the 306 MEG sensor measurements forming the multivariate pattern at each time point (e.g., Figures 2A and 2B). For stimulus decoding, each trial’s label was the stimulus identity (i.e., the continuum step number, from 1 to 10), and we conducted pairwise classification of each of the 45 possible stimulus pairings. For percept decoding, each trial’s label came from the participant’s response on that trial (i.e., *ba* or *da*), and we conducted binary classification of all trials labeled *ba* vs. all trials labeled *da*. Thus, in all cases, the classifier was trained to distinguish two conditions.

For cross-validation, the data were randomly assigned to one of five folds; four folds were used for training the classifier and one fold was used for testing it. The data were then equalized in noise level using the “epoch” method of multivariate noise normalization (Guggenmos et al., 2018), which computes the noise covariance matrix for all time points in the epoch separately within each condition and then averages across time points and conditions. The noise covariance was estimated from the training data and then applied to both the training and the test data, which avoids inflated classification. Further, because the estimate of noise covariance may be unstable when there are relatively few data points with respect to the number of features, we used the shrinkage transformation (Ledoit & Wolf, 2004) to regularize the estimate and thus prevent overfitting. To reduce computational load and improve the signal-to-noise ratio, trials corresponding to the same condition within each of the five folds were averaged together, yielding one summary trial for each condition per fold. Thus, for stimulus decoding, each summary trial reflected eight real trials, and for percept decoding, each summary trial reflected ∼40 real trials, depending on the proportion of *ba* vs. *da* labels applied by the participant during the 400 experimental trials. The final decoding accuracies reflected the average over 100 repetitions of the entire procedure. In all cases, 50% represents chance performance of the classifier, and higher decoding accuracies reflect greater neural dissimilarity.

In order to determine whether information about stimulus identity and/or perceptual label is lateralized, we also performed SVM decoding separately on left-hemisphere sensors (*n* = 144) and right-hemisphere sensors (*n* = 144) as described above. Magnetometers and gradiometers at the midline (*n* = 18) were excluded from these two subgroups.

### Statistical Testing

We used nonparametric permutation tests to determine significance in decoding-accuracy time courses. All time points in the trial (−200 to 1,000 ms; 1-ms resolution) were included in each test. The null hypothesis was equal to the 50% chance level. Under the null hypothesis, the subject-specific time courses can be randomly flipped around their null values before averaging across subjects to yield permutation samples. Repeating this procedure 5,000 times enabled us to estimate the empirical distribution of the decoding-accuracy values and convert the original time courses into *p*-value maps. Finally, the familywise error across time points was controlled using cluster-size inference. Namely, suprathreshold clusters (i.e., contiguous time points) were identified by applying a cluster-defining threshold of *p* = 0.05. These clusters were then reported as significant if their length in time exceeded a *p* = 0.05 threshold (the 95th percentile) with respect to the empirical distribution of the suprathreshold clusters of the permutation-sample statistical maps. These statistical tests were one-sided to reflect hypotheses about the direction of effects.

### Transformed Classifier Weights

To support the lateralization analysis, we identified which sensors contributed to the decoding of *ba* vs. *da*. Multivariate classifiers, such as the linear SVM that we used, combine information across sensors and weight them according to their contribution to distinguishing the labeled input. No single weight, however, is directly interpretable because it is only in combination that they produce the extraction filter that best amplifies signal and suppresses noise (Haufe et al., 2014). We used the method described by Haufe and colleagues (Haufe et al., 2014) to transform the weights into intuitive and neurophysiologically interpretable values for each sensor. Briefly, to apply this transformation, the weight matrix was left-multiplied by the data covariance and right-multiplied by the inverse of the latent factors’ covariance. This transformation yields values that can be interpreted as neural activations that encode the discrimination of *ba* vs. *da*. The transformed classifier weights were then averaged across participants and displayed on a topographical sensor map for a visualization of the spatiotemporal origin, direction, and strength of the *ba* vs. *da* neural signal. The MEG helmet has 102 sensor locations, each containing one magnetometer and two planar gradiometers. We plotted the magnetometer and gradiometer values separately because the two sensor types capture different aspects of the magnetic field: Magnetometers measure the component of the magnetic field that is perpendicular to the surface of the helmet (with more sensitivity to distant sources) and planar gradiometers estimate the spatial derivative of that component (with less sensitivity to distant sources).

### Representational Similarity Analysis

We used RSA to reveal how information about stimulus identity is structured in the MEG data. In the RSA framework, the neural RDM derived from classifier performance is correlated with RDMs representing hypothesized models of stimulus dissimilarity. Each cell of a 10 × 10 symmetric RDM contains the dissimilarity of the row and column stimuli. We tested two models: a Perceptual model and an Ambiguity model.

The Perceptual RDM was created by averaging across participants’ behavioral responses in the Active task. Each cell contained the absolute value of the difference between the percent of trials on which the column stimulus was labeled *ba* and the percent of trials on which the row stimulus was labeled *ba*, yielding a matrix with minimum value 0 and maximum value 100.

The Ambiguity RDM was created by comparing the consistency of participants’ responses to each stimulus. The proportion of *ba* responses was first transformed into a consistency index, where 100 indicated that the stimulus was given the same label on every trial (i.e., consistent and therefore unambiguous) and 0 indicated that it was labeled *ba* on exactly 50% of trials (i.e., inconsistent and therefore ambiguous). Each cell of the matrix was then populated with the absolute value of the difference between the consistency of the column stimulus and the consistency of the row stimulus. Thus, this matrix, also averaged across participants, represents the dissimilarity of stimulus pairs in terms of their perceptual ambiguity: Low values indicate that both stimuli are either ambiguous or unambiguous; high values indicate that one stimulus is ambiguous and the other is unambiguous.

We used a bootstrapping procedure for statistical inference in RSA. Bootstrap samples were created by resampling subjects 5,000 times with replacement. For each bootstrap sample, we created subject-averaged, time-resolved neural RDMs for the Passive task and the Active task and compared them with the resampled Perceptual model and the resampled Ambiguity model, using partial Spearman correlation in order to partial out the other model. These correlation values were used to construct statistics representing the main effect of task, the main effect of model, and the interaction of task and model. The empirical distribution of these statistics over the bootstrap samples enabled the estimation of *p* values by assessing the percent of bootstrap samples crossing the 0 value while accounting for two-sided hypothesis tests. Because we ultimately conducted these bootstrap percentile tests in the three time windows identified during *ba* vs. *da* decoding, we assessed significance with respect to the Bonferroni-corrected *α* level of 0.017.

### Multidimensional Scaling and *k*-Means Clustering

In order to visualize the structure of the neural dissimilarity data, we performed nonmetric multidimensional scaling (MDS) on the time-resolved neural dissimilarity matrices using an 80-ms sliding window with a 20-ms step (cf. E. F. Chang et al., 2010) and 10 randomly chosen initial configurations for each scaling procedure. The goodness-of-fit criterion was Kruskal’s “stress 1” (Kruskal, 1964). *k*-means clustering was performed using the squared Euclidean distance metric. All analyses were performed in MATLAB (mathworks.com).

## RESULTS

### Behavioral Responses During the Passive Task

Behavioral responses were collected in the Passive task to ensure arousal and discourage attention to the auditory stimuli. For the target trials in the visual cover task, which were excluded from neural analysis, participants (*n* = 23) achieved a mean hit rate of 0.98 (*SD* = 0.04; range = 0.87–1) with a mean response time of 505 ms (*SD* = 95; range = 340–695). One additional participant was observed via video feed to be making appropriate motor responses during this task, but no button-presses were recorded; this participant was included in all subsequent analyses for a total of *n* = 24.

### Behavioral Responses During the Active Task

Labeling responses were collected from participants (*n* = 24) in the Active task to assess categorical perception of the stimulus continuum. Stimuli 1–5 were primarily labeled *ba* and stimuli 6–10 were primarily labeled *da* (Figure 2C). Substantial ambiguity in identifying steps 5 and 6 is consistent with the original identification data reported for these stimuli (Stephens & Holt, 2011).

### Decoding the *ba* vs. *da* Percept in the Active Task

We first decoded the category labels applied during the Active task from participants’ individual brain responses measured at all 306 MEG sensors. Figure 2D (black line) shows the time course of decoding accuracy averaged across participants; decoding was significantly above chance (one-sided sign-permutation test; *p* < 0.05 cluster-defining threshold; *p* < 0.05 cluster threshold) during the three time windows, 165–354 ms (*p* = 0.007), 429–529 ms (*p* = 0.042), and 569–680 ms (*p* = 0.034). These results indicated that the MEG data contained sufficient information to decode an individual’s subjective, categorical perception of an acoustic continuum during the Active task.

We next examined hemispheric contributions to decoding *ba* vs. *da* perception. We repeated the decoding analysis, this time using only left-hemisphere sensors. These decoding-accuracy results (Figure 2D, solid gray line) essentially recapitulated the time course of decoding using all sensors (black line). Left-hemisphere decoding was significantly above chance in one time window, 156–350 ms (*p* = 0.005). We also performed decoding using only right-hemisphere sensors: these results (dotted gray line) were similar in latency and shape to the others, but did not reach significance. Lastly, we performed three one-sided sign-permutation tests (all > left, all > right, left > right) to determine whether these three traces were significantly different from one another: No differences were identified.

To confirm lateralized contributions to *ba* vs. *da* decoding, we examined the spatial distribution of transformed classifier weights (Haufe et al., 2014). The values projected on the topographical maps can be interpreted as neural activations that encode the *ba* vs. *da* distinction measured at magnetometers (Figure 2E) and planar gradiometers (Figure 2F). For visualization, we averaged the transformed weights within each of the three time windows during which all-sensors decoding was above chance (from Figure 2D). In the first time window, the strongest signal was located in left-temporal sensors, which corroborated the significant left-hemisphere decoding at this latency. In the second time window, the signal was also left-dominant. In the third time window, the patterns were bilateral, without a clear hemispheric dominance. Together, the decoding results and transformed classifier weights indicated that neural responses in the left hemisphere provided the first wave of reliable perceptual information, and that the right hemisphere was necessary but not sufficient for subsequent reliable percept decoding.

In the Passive task, stimulus tokens were not given a perceptual label, so the binary *ba* vs. *da* classification could not be performed on these data. Instead, we next conducted a head-to-head comparison of how Passive vs. Active task demands affected the neural representations of the 10 continuum stimuli.

### Strength and Maintenance of Stimulus Information in Passive vs. Active Tasks

To determine when patterns of neural activity at the sensor level distinguish continuum stimuli from one another, we averaged all pairwise decoding accuracies from the neural RDMs at each time point separately, yielding a time series of overall stimulus decoding accuracy for each participant and each task. If the demands of the Active task increase the overall dissimilarity of stimulus representations, we should observe significantly higher overall decoding accuracy in the Active vs. the Passive task.

**Figure 3.**
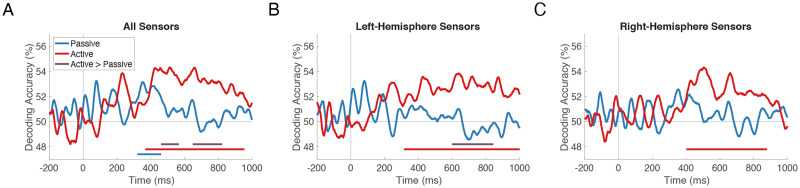
Strength and maintenance of stimulus information in Passive vs. Active tasks. The time course of overall stimulus decoding accuracy, averaged across all pairwise comparisons and participants, is shown for the Passive (blue) and Active (red) tasks. Blue and red horizontal lines indicate when the corresponding time series is above chance. Purple lines indicate time windows in which decoding accuracy is significantly higher in Active vs. Passive. Decoding was performed in (A) all sensors, (B) left-hemisphere sensors, and (C) right-hemisphere sensors, with similar results in each.

The onset of significant decoding occurred at 321 ms in the Passive task and at 368 ms in the Active task; overall stimulus decoding remained above chance (one-sided sign-permutation test; *p* < 0.05 cluster-defining threshold; *p* < 0.05 cluster threshold) for 140 ms in the Passive task (Figure 3A, blue line; *p* = 0.017) and for 586 ms in the Active task (red line; *p* < 0.001). Overall stimulus decoding was reliably higher in the Active vs. the Passive task (one-sided sign-permutation test; *p* < 0.05 cluster-defining threshold; *p* < 0.05 cluster threshold) in two time windows between 461 and 823 ms (purple lines; *p* = 0.037 and *p* = 0.010). These windows began at the offset of above-chance Passive decoding and coincided with times of above-chance Active decoding, suggesting that Active vs. Passive stimulus representations were not necessarily stronger at any given time, but were maintained longer in the Active task.

In order to determine whether these task effects differed by hemisphere, we performed the same tests on decoding results obtained from left- and right-hemisphere sensors separately. In the left hemisphere (Figure 3B), Active stimulus decoding was above chance from 315 to 1,000 ms (*p* < 0.001); Passive stimulus decoding was not above chance; and there was a significant difference between these two time courses (600–839 ms; *p* = 0.002). In the right hemisphere (Figure 3C), Active stimulus decoding was above chance (402–878 ms; *p* < 0.001); Passive stimulus decoding was not; and the difference between these two time courses was not significant. Thus, maintenance of stimulus information over time in Active trials was observed regardless of whether decoding was performed in all, left-hemisphere, or right-hemisphere sensors.

However, while reliable overall stimulus decoding indicates that stimulus information is present in the data, it does not reveal the *structure* of that information. The higher overall decoding accuracy in the Active task could be due to a nonselective boost in the stimulus-related signal, perhaps as a consequence of attention. On the other hand, it could be due to selective increases in dissimilarity for certain pairs of stimuli. Therefore, we next interrogated the dissimilarity structure of these neural stimulus representations over the course of Passive and Active trials.

### Structure of Stimulus Information in Passive vs. Active Tasks

A second goal of our study was to describe the dissimilarity structure of the neural data over time and as a function of task demands. The premise of these analyses is that the structure of continuum representation can be inferred from the classifier’s ability to discriminate stimuli along it. We expected that, over time, neural dissimilarities would converge on two perceptual categories, *ba* and *da*. One hypothesis was that this pattern would be more evident in the Active task, where a delayed categorical response was required. Alternatively, if speech-continuum tokens undergo an obligatory transformation into phonemes, this pattern might be observed in both tasks.

First, we analyzed specific pairs of stimuli that exemplified within-category and between-category contrasts in order to test the hypothesis that the demands of the Active task warp the neural representation of the acoustic continuum in favor of phoneme categories. Second, we used RSA to determine whether and when the neural representation of the continuum was better explained by models of perceptual categories vs. stimulus ambiguity. Third, we applied a clustering algorithm to the neural dissimilarities.

#### Within-Category and Between-Category Comparisons

We borrowed an approach from traditional categorical-perception studies and compared the neural dissimilarity of stimulus pairs that were equidistant from each other on the acoustic stimulus continuum, but fell within vs. across the phoneme category boundary. Based on the behavioral results from the Active task (Figure 2C), the boundary was presumed to lie between stimulus 5 and stimulus 6. We therefore selected stimuli 1 and 4 as the within-*ba* pair, stimuli 4 and 7 as the between-category pair, and stimuli 7 and 10 as the within-*da* pair. If the task demands of Active categorization decrease the dissimilarity of within-category neural representations, we should observe significantly higher within-category decoding accuracy in the Passive task than in the Active task. If the task demands of Active categorization increase the dissimilarity of between-category neural representations, we should observe significantly higher between-category decoding accuracy in the Active task than in the Passive task.

Using the pairwise stimulus decoding results from when the classifier was built and tested on data from all sensors, we isolated the decoding accuracy time series for the within-*ba* pair and ran a one-sided sign-permutation test with cluster-size correction for Passive > Active. No significant clusters were identified by this procedure. An identical procedure was followed for the within-*da* pair; no significant clusters were identified. For the between-category pair, the data were submitted to the same test for Active > Passive, and no significant clusters were identified. Thus, we found no evidence of task demands affecting the dissimilarity of these acoustically equidistant within-category or between-category neural patterns at any point in the trial.

#### Representational Similarity Analysis

**Figure 4.**
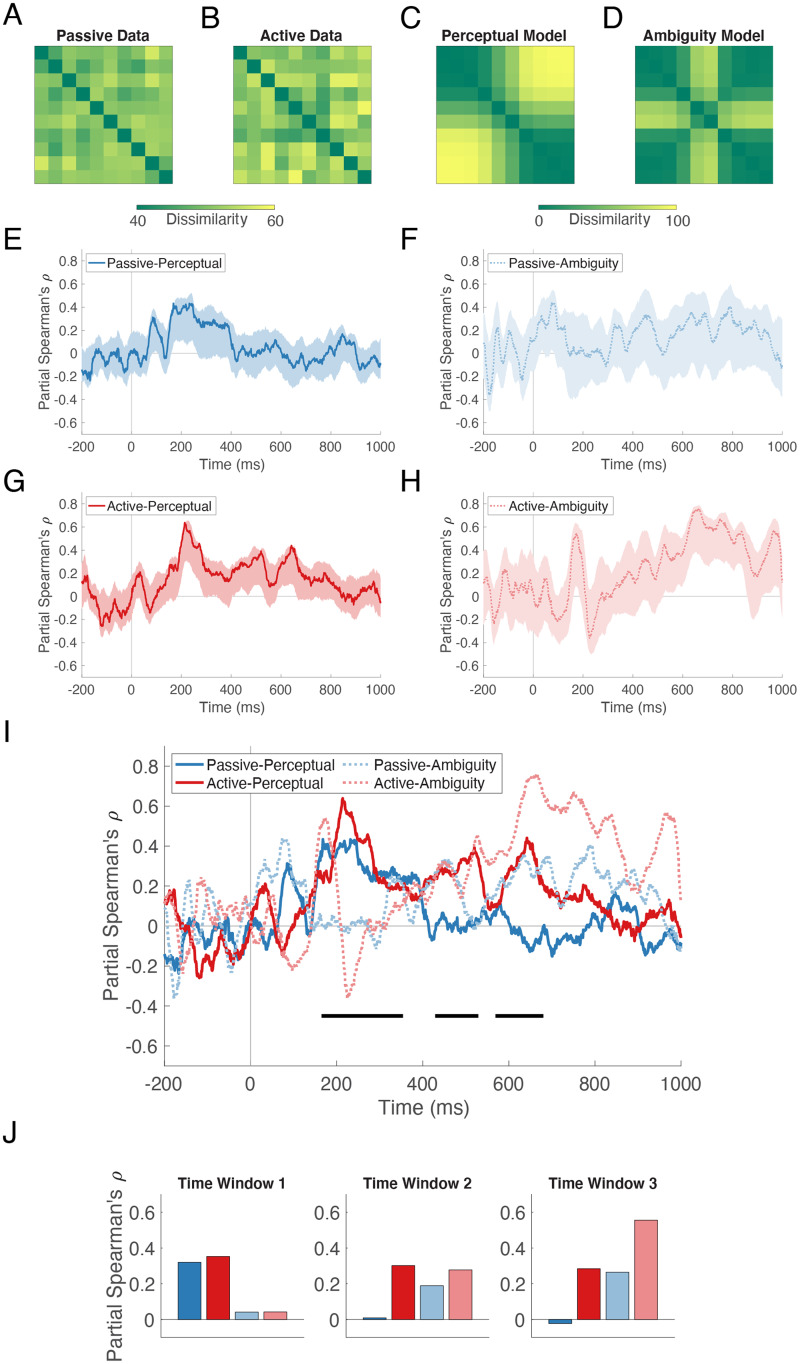
Structure of stimulus information revealed by representational similarity analysis. Example representational dissimilarity matrices at 130 ms (peak amplitude of the auditory evoked field, per Figures 2A and 2B) for the Passive (A) and Active (B) tasks. (C) Perceptual dissimilarity model. (D) Ambiguity dissimilarity model. For RSA, the average neural dissimilarity matrix for each task at each time point was correlated with each model matrix, yielding four neural-model correlation time series (E, F, G, H), each plotted with its 95% confidence interval. For easier comparison, all four traces are plotted together in (I). Horizontal lines below the traces indicate the time windows of significant *ba* vs. *da* decoding from Figure 2D, within which we tested for differences in correlation coefficients using the bootstrap percentile method. In the first time window, there was a significant effect of model, and in the third time window there were significant effects of model and task. (J) Extracted correlation coefficients show that the Perceptual model was superior in the first time window and the Ambiguity model was superior in the third time window. Additionally, in the third time window, neural-model correlations were higher in the Active task than in the Passive task. Note that in Figure 4, all neural data come from stimulus decoding performed on all sensors.

The dissimilarity structure of the neural data can be visualized in a time-resolved matrix in which each cell contains the pairwise decoding accuracy of the row and column stimuli in the Passive task (Figure 4A) and the Active task (Figure 4B). In order to determine if and when the neural data is structured by phoneme perception and/or stimulus ambiguity, we constructed two models based on participants’ labeling responses in the Active task (Figure 2C).

The Perceptual model (Figure 4C) was derived from the proportion of *ba* vs. *da* labels. If perception is represented in the neural data, then two stimuli that are usually given the same label will be poorly classified (low dissimilarity), while two stimuli that are usually given different labels will be well classified (high dissimilarity).

The Ambiguity model (Figure 4D) reflected the degree to which those labels were consistently applied across trials, and specifically whether two stimuli differed in this regard. If differences in ambiguity are represented in the neural data, then two stimuli that are acoustically different but both unambiguous (e.g., 1 and 10) will be poorly classified (low dissimilarity); two stimuli that are acoustically similar and both ambiguous (e.g., 5 and 6) will be poorly classified (low dissimilarity); and two stimuli that differ in ambiguity (e.g., 1 and 5) will be well classified (high dissimilarity).

We then computed the partial Spearman correlation between each task’s neural RDM and the two candidate model RDMs, partialling out the other model. The outcome was a time course of neural-model representational similarity, in which a higher correlation indicates greater similarity. The four RSA correlation time series and their bootstrapped 95% confidence intervals are presented for qualitative intuition in Figure 4. The Passive-Perceptual correlation reached a brief plateau around 200 ms and subsequently decayed (Figure 4E). The Passive-Ambiguity correlation peaked transiently at 74 ms (Figure 4F). The Active-Perceptual correlation had three peaks, at 214, 520, and 642 ms (Figure 4G). The Active-Ambiguity correlation peaked initially at 173 ms and then remained reliably positive between 521 and 859 ms (Figure 4H).

For the statistical analysis of model fit, without strong justification for any a priori time windows of interest, we selected the three time windows within which *ba* vs. *da* was robustly decoded (from Figure 2D). As can be seen in Figure 4I, where all four traces are displayed together, peaks in the correlation time series align well with these time windows (horizontal black lines). Within each window, we extracted the mean bootstrapped correlation coefficient and tested for a main effect of task, a main effect of model, and a task × model interaction using the bootstrap percentile method.

In the first time window (165–354 ms), there was a significant effect of model (*p* = 0.016). In the second time window (429–529 ms), no effects were significant (*p*’s > 0.132). In the third time window (569–680 ms), there was a significant effect of model (*p* = 0.006) and a significant effect of task (*p* = 0.002). The extracted mean correlation coefficients (partial Spearman’s *ρ*) in Figure 4J show that the Perceptual model was a better fit in the first time window, and that the Ambiguity model was a better fit in the third time window. Additionally, neural-model correlations were higher in the Active task than in the Passive task in the third time window.

RSA conducted on the neural decoding results by hemisphere produced similar but weaker results (data not shown). In the left hemisphere, there was an effect of task in the third time window (Active > Passive; *p* = 0.003). In the right hemisphere, there was an effect of model in the third time window (Ambiguity > Perceptual; *p* = 0.011).

#### Multidimensional Scaling and k-Means Clustering

RSA revealed that as correlations with the Perceptual model waned over time, robust correlations with the Ambiguity model emerged. We confirmed these results by visualizing the neural dissimilarities in a common geometric space and then implementing a clustering algorithm. The closer two stimuli are in this space, the more similar their neural patterns are; If a categorical representation is present in the data, we should see a spatial clustering of stimuli belonging to that category.

We applied MDS to the time-resolved dissimilarity matrices averaged over participants. In selecting the number of dimensions to specify, we confirmed that Kruskal stress decreased as the number of specified dimensions increased, and that this pattern held for both Passive and Active data as well as at different time points in the trial interval (data not shown). Because stress values for the two-dimensional solution consistently fell below 0.2, which indicated an adequate fit (Kruskal, 1964), we used this embedding in the subsequent clustering analysis.

**Figure 5.**
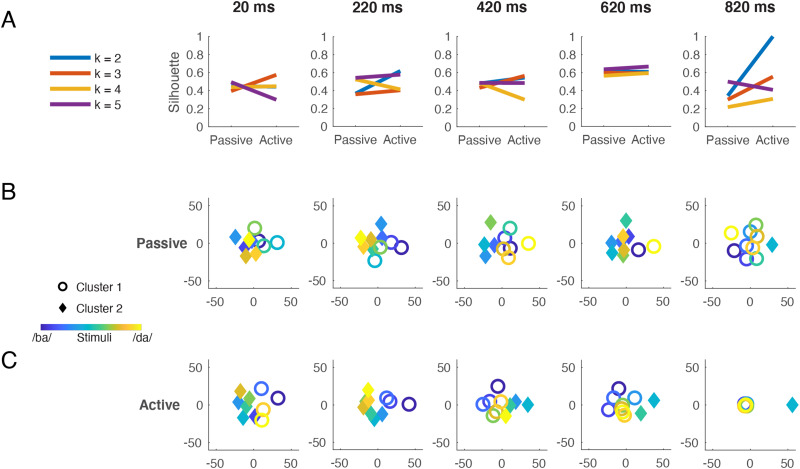
Structure of stimulus information revealed by multidimensional scaling and *k*-means clustering. Neural dissimilarities from the Passive and Active tasks were scaled to two dimensions using multidimensional scaling and the resulting distances were submitted to *k*-means clustering. (A) Silhouette values indicate how many clusters optimally describe the data. Little difference is observed between Passive and Active when three, four, or five clusters are imposed. A two-cluster solution better fits the Active than the Passive data toward the end of the trial interval. The two-cluster solution (i.e., *k* = 2) is plotted at five representative time points throughout the trial interval for Passive (B) and Active (C) data. The stimulus continuum is represented by color: from blue (/ba/) to yellow (/da/). Cluster membership is represented by shape: circles vs. diamonds; in each plot, circles are used for the cluster to which stimulus 1 (/ba/ endpoint) is assigned. The time stamp indicates the center of an 80-ms analysis window. In the lowest far-right plot (Active, 820 ms), circles have been jittered for visibility. At 220 ms, both tasks show a phonemic patterning of *ba*’s (blue circles) vs. *da*’s (yellow diamonds). At subsequent time points, both tasks show a patterning of category goodness (blue and yellow circles) vs. ambiguity (green diamonds). Note that in Figure 5, all neural data come from stimulus decoding performed on all sensors.

To formally determine how the neural data cluster, we applied *k*-means clustering, with different values of *k*, to the MDS two-dimensional solution. In order to validate cluster membership, the average silhouette value, a measure of within-cluster tightness and between-cluster separation (Rousseeuw, 1987), was calculated for each time-resolved clustering solution. Consistent with the top-down task-demands hypothesis, the two-cluster solution was notably better for the Active data than for the Passive data near the end of the trial interval (Figure 5A). In contrast, silhouette values for three, four, and five clusters were very similar for Passive and Active data, indicating little effect of task on item clustering at these values of *k*.

We then examined which representations were assigned to the two clusters over time, under the hypothesis that the two clusters that best fit the data at the end of the trial would correspond to the *ba* and *da* phoneme categories. Figures 5B and 5C plot the cluster assignments (circles vs. diamonds) for each of the 10 stimuli (blue/*ba*-yellow/*da* continuum) at five representative time points throughout the trial. The analysis window centered on 220 ms encompasses peak *ba* vs. *da* decoding accuracy (235 ms, from Figure 2D) and peak Active-Perceptual correlation (214 ms, from Figure 4I); at this latency, clusters in both Passive and Active data reflected *ba* (blue circles) and *da* (yellow diamonds) categories. At later time points, cluster composition reflected a segregation of endpoint tokens (blue and yellow circles) from midpoint tokens (green diamonds). Near the end of the trial, the ambiguous stimulus 5 (from Figure 2C) was placed in its own cluster. Thus, the neural data from both tasks clustered according to phoneme percept at ∼220 ms, but represented stimulus ambiguity, or a lack thereof, for a subsequent ∼600 ms. This late emergence of an ambiguity representation is consistent with the RSA results described above.

## DISCUSSION

### Summary of Results

Speech perception relies on the interpretation of acoustically variable phoneme realizations, a process so efficient that it mostly escapes our notice in the course of everyday listening. We found that patterns of activity in MEG sensors could be used to decode whether participants perceived *ba* or *da* as they categorized tokens from a 10-step /ba/-/da/ continuum. The left hemisphere was sufficient to decode the percept early in the trial, while the right hemisphere was necessary but not sufficient for decoding at later time points.

We also decoded the individual stimuli from the neural patterns evoked during active categorization of and passive exposure to these stimuli. We found that, in general, stimulus information was maintained longer when a response was required. To understand how that information was structured, we examined the neural dissimilarities across the continuum, finding a *lack* of evidence for the loss of within-category detail during the categorization task. We also found evidence *for* the retention of subphonemic detail by examining how two models of stimulus dissimilarity fit the data within the three time windows during which *ba* vs. *da* decoding was above chance. The Perceptual model, which distinguishes stimuli that are given different labels, was superior in the earliest time window. The Ambiguity model, which distinguishes stimuli that are consistently vs. inconsistently labeled, was superior in the latest time window. Thus, even as a categorical phoneme representation was present in the data, subphonemic information about stimulus ambiguity had not been discarded.

### Lateralized Contributions to Categorical Perception

We examined whether the information supporting *ba* vs. *da* decoding was left-lateralized, as might be expected from previous neuroimaging studies of categorical speech perception (Alho et al., 2016; Altmann et al., 2014; Joanisse et al., 2007; Y. S. Lee et al., 2012; Myers et al., 2009). First, we simply repeated the classification of neural activity patterns measured at all sensors, but this time restricting the classifier to only left-hemisphere sensors or only right-hemisphere sensors. Reliable decoding of the *ba* vs. *da* percept was attained using all sensors and left-hemisphere sensors. However, all three analyses had similar time courses of decoding accuracy and were not significantly different from one another. Our right-hemisphere results are consistent with the theory that the right hemisphere holds non-categorical acoustic representations which are suboptimal but sufficient for speech perception (Hickok & Poeppel, 2007). Second, we examined the transformed classifier weights that contributed to decoding performance when the classifier had access to all sensors, finding that the signal was first present in the left hemisphere, and subsequently in both hemispheres. This is consistent with a meta-analysis of functional brain imaging in healthy right-handed participants whose authors concluded that the right hemisphere does not itself hold phonological representations, but participates in inter-hemispheric language processing (Vigneau et al., 2011).

Both the stimulus type (a place-of-articulation continuum) and the task (phonological analysis) favor the involvement of the left hemisphere. The syllables /ba/ and /da/ are distinguished by the placement of the tongue during articulation of the initial consonants, which manifests acoustically as a rapid upward vs. downward transition into the vowel’s second formant. Perceiving this transition requires fine-grained temporal processing, which has been argued to be a specialty of the left hemisphere (Arsenault & Buchsbaum, 2015; Poeppel, 2003; Zatorre & Belin, 2001). Explicit phonological analysis of isolated, sublexical speech sounds is generally performed in the left hemisphere (Turkeltaub & Coslett, 2010), but the right hemisphere may have this capacity as well (Hickok & Poeppel, 2000; Poeppel et al., 2004). For example, fMRI patterns in right temporal cortex have been used to decode participants’ trial-by-trial perceptions of ambiguous speech sounds (Luthra et al., 2020).

### Decision-Related Maintenance of Stimulus Information

We next turned to decoding the 10 stimuli instead of the binary percept. Averaging across all pairwise decoding accuracies provided a coarse measure of the strength and maintenance of neural stimulus representations in the Passive and Active tasks. As hypothesized, stimulus information was maintained longer in the Active task. Significant decoding peaked transiently around 400 ms in the Passive task and extended to nearly 1,000 ms in the Active task. This finding was consistent with the main effect of task identified in the RSA, in which neural-model correlations were higher in the Active task in the third time window (569–680 ms). To begin to explain these results, we note that, in contrast to the Passive task, the Active task required attention to the auditory stimuli, a decision, and, unavoidably, a motor response. We aimed to reduce the influence of preparatory motor activity on the classifiers by counterbalancing the finger-response pairings within each participant such that each finger was not uniquely associated with *ba* or *da*; the fact that *ba* vs. *da* decoding fell to chance at 680 ms instead of continuing until 900 ms when a response was allowed suggests that this strategy was successful (Heekeren, Marrett, & Ungerleider, 2008).

In human brain imaging, MVPA of attended vs. unattended stimuli has been shown to yield better classification performance (Bugatus et al., 2017). Direct neural recordings in primary auditory cortex demonstrate that, as compared to passive exposure, performing a detection task boosts the correlated activity of neurons with similar tuning, yielding an enhancement of population coding (Downer et al., 2015). Although we anticipated that similar enhancements throughout perisylvian cortex would contribute to overall decoding accuracy in the Active task, overall Active decoding was not significantly higher than overall Passive decoding in the short period around 400 ms during which both were above chance. However, it must be noted that the Active task was not a detection task, nor did it direct attention to subphonemic detail per se (e.g., by requiring discrimination). Thus, we can only conclude that *general* attention did not modulate stimulus representation at this latency.

Alternatively, it could be the case that, by averaging over all pairwise decoding accuracies, some increases in dissimilarity cancelled out other decreases in dissimilarity, obscuring effects of attention on subsets of the representational matrix. We addressed this issue by examining specific pairs of stimuli that exemplified between- and within-category dissimilarities, although this too yielded no apparent effect of task. This result is not consistent with a study conducted in mice in which auditory cortex showed significant shifts in frequency tuning at both the neural and population level during the performance of active sound categorization vs. passive exposure (Xin et al., 2019). In that study, the active task was characterized by enhanced representation of stimuli near the boundary, and pairwise classification at the population level showed increased dissimilarity for between-category stimuli and decreased dissimilarity for within-category stimuli in the active vs. passive condition. Human behavior, too, reveals acquired similarity (i.e., reduced behavioral discrimination) for tokens within an arbitrary auditory frequency category once subjects are trained to categorize them (Guenther et al., 1999). Despite finding no evidence to support such prior reports and our own hypothesis of more categorical patterns under active conditions, our results do affirm the representation of speech information in the brain during diverted attention. Both tasks showed strong correlations with the Perceptual model in the first time window (165–354 ms), suggesting that speech processing occurs with high fidelity even when it is passively encountered.

The third variable that differentiates the Passive and Active tasks is the decision, and so the maintenance of stimulus representations in the Active task might be attributed to decision-related processes. Neural correlates of decision processes (unique to the Active condition) can be distinguished from those of sensory processes (shared by Passive and Active conditions), as the former vary with the difficulty of the perceptual decision due to the need to accumulate evidence until a decision criterion is met (Binder et al., 2004). Maintenance in the Active task might then reflect working memory in the service of evidence accumulation (Curtis & Lee, 2010). Previous studies confirm that auditory working memory is both measurable in MEG (Huang et al., 2016) and able to maintain precise subphonemic detail for a single speech sound without conversion to an abstract phoneme (Joseph et al., 2015). Due to the 900-ms enforced delay between stimulus onset and permitted button-press, it is likely that our neural data also captured post-decision processing (i.e., while participants waited to respond), including metacognitive monitoring and/or continued evaluation of the sensory evidence (Yeung & Summerfield, 2012). Although the Active task itself was simple, the middle tokens were, empirically, ambiguous, and participants may have had low confidence in their decisions on those trials. This is a particularly compelling interpretation given the evidence from RSA and clustering that stimulus information about ambiguity dominated the latter half of Active trials. Therefore, we attribute the prolonged stimulus decoding in the Active task (368–954 ms), as well as the Active > Passive effect revealed by RSA in the third time window (569–680 ms), to decision-related, potentially metacognitive, processes.

### Subphonemic Representations of Ambiguity

In the brain’s quest for meaning, speech sounds are subject to interpretation. With multivariate pattern analysis of fMRI data it is possible to recover the subjective interpretation of such stimuli (Bonte et al., 2017; Kilian-Hutten et al., 2011; Luthra et al., 2020). Here we find that MEG patterns can also be used to decode the categorical labels that participants apply to stimuli from a /ba/-/da/ continuum. The onset of significant category decoding in our study (165 ms) is consistent with other reports of phoneme-category effects between 110 and 175 ms (Bidelman et al., 2013; E. F. Chang et al., 2010; Toscano et al., 2018).

By comparing the dissimilarity structure of stimulus representations over time and across tasks, we attempted to determine whether the emergence of a categorical phoneme representation is an obligatory part of bottom-up speech processing, or whether it is task-dependent. We reasoned that the outcome of the Active task’s decision process was certain to be a phoneme, but that, under passive listening conditions, neural representations might also converge on a “report-independent” abstraction of the sensory evidence (Freedman & Assad, 2011, p. 145). Instead, using RSA, we found that the data from both tasks were well described first by the Perceptual model and later by the Ambiguity model. The clustering analysis confirmed that both tasks’ scaled neural dissimilarities reflected phonemes in the first half of the trial interval and ambiguous vs. unambiguous tokens in the second half. This suggests an obligatory representation of ambiguity during speech processing, regardless of task. Such a result is perhaps inconsistent with the subjective experience of categorical perception in which, when a sequence of gradually morphed sounds (e.g., from /ba/ to /da/ in 10 steps) is played aloud, the listener reports an abrupt change from the *ba* percept to the *da* percept, rather than segues from *ba* to *ambiguous* and from *ambiguous* to *da*. This raises the question of whether the neural “tag” of ambiguity exists independently of the phonemic interpretation of the stimulus that rises to the level of conscious awareness (see Davis & Johnsrude, 2007, for a discussion).

The literature on a higher level of speech processing—spoken word recognition—lends support to this possibility. While the present study was concerned with speech perception in isolation, spoken word recognition involves segmenting a stream of input and interfacing with the mental lexicon; presumably, the purpose of speech perception is to allow spoken word recognition (Samuel, 2011). Spoken word recognition is mediated by top-down influences such as lexical knowledge (Brodbeck et al., 2018), semantic context (Broderick et al., 2019), and talker information (Nygaard & Pisoni, 1998), which establish expectations about the form and content of upcoming speech. Ambiguous input, such as a noise-masked phoneme, tends to be interpreted in line with those expectations (Leonard et al., 2016; Warren, 1970). Such contextual influences were beyond the scope of this paper, but recent work indicates that in the absence of a constraining preceding context, a marker of ambiguity can also be carried *forward* in time, where it facilitates reanalysis once disambiguating information becomes available (Gwilliams et al., 2018). Maintaining subphonemic detail for a second or more (Connine et al., 1991; Szostak & Pitt, 2013) may be an adaptive strategy, aiding recovery from an initial interpretation of the speech signal that is subsequently revealed to be incorrect (McMurray et al., 2009).

Of course, this phenomenon does not preclude a parallel process in which an initial commitment to one phoneme is made (Gwilliams et al., 2018). This position is consistent with models of perceptual decision-making not specific to speech that posit a functional module for detecting uncertainty or difficulty that operates alongside the perceptual cascade (Heekeren et al., 2008) or allow for storage of both the categorical decision and the information on which it was based (Lemus et al., 2007). In speech perception, some ascending neural representations may be transformed into those indicative of category membership, giving rise to the neural correlates of categorical perception (e.g., Joanisse et al., 2007; Liebenthal et al., 2005; Myers et al., 2009), while another processing stream may retain subphonemic detail (e.g., Toscano et al., 2010, 2018), which is very often systematically informative to the listener (Hawkins, 2003). Word segmentation (Smith & Hawkins, 2012), compensation for coarticulation (Mann, 1980), and talker identification (Remez et al., 1997) are just some of the fundamental perceptual abilities that rely on phonetic detail. Furthermore, it is believed that speech perception cannot involve complete abstraction, because subphonemic information is retained in listeners’ word-specific and even episodic phonetic memories of speech (Goldinger, 1996; Pierrehumbert, 2016).

Our findings, in which neural representations of stimulus ambiguity and categorical percept were identified within the same time window, are consistent with the notion of parallel streams. Thus it may be the case that subphonemic details and abstract phonemes may be represented, and therefore decoded, simultaneously (Diesch et al., 1996), and that complementary representations may balance efficiency in recognizing spoken words with flexibility in responding to shifting task demands and goals. To speculate further, an accompanying tag of ambiguity may be related to feedback or prediction-error signals that guide perceptual retuning (Sohoglu & Davis, 2016).

### Limitations and Future Directions

This work has made an incremental but novel contribution to the study of categorical speech perception by using time-resolved neural dissimilarities to index the representation of an acoustic continuum across tasks. There are some limitations to this approach, however. For one, the absence of significant effects in the comparison of within-category and between-category neural dissimilarities cannot be interpreted as evidence that the brain represents the continuum stimuli in the same way across tasks. It is difficult to rule out the possibility that true-positive signals could not be detected with MEG because there is no straightforward way to link multivariate decoding accuracies with standard effect size measures (Hebart & Baker, 2018).

A second caveat is that we tested only a single stimulus continuum, and our results may not generalize to the decoding of other consonant or vowel continua due to differences in how their spectral and temporal properties are represented in the brain. The generalizability of these findings is itself a worthy question because the sounds of one’s native language are so highly overlearned. For example, we found a robust neural representation of the perceptual structure of the continuum even when participants’ attention was diverted—would this occur for non-linguistic stimuli (Bidelman & Walker, 2017) or in a language being learned? A longitudinal study could perhaps use multivariate decoding to document any corresponding neural changes in representational structure as participants acquire new phoneme categories or expertise in a new perceptual domain.

Another question inspired by our findings is whether the ambiguity effect is due to a lower-order or a higher-order property of the stimuli. That endpoint tokens cluster together suggests a higher-order representation of prototypicality independent of acoustics; however, very early ambiguity effects have also been interpreted as lower-order sensory information related to distance from the phoneme boundary (Gwilliams et al., 2018). We observed early spikes in both tasks’ Ambiguity correlations prior to the sustained late Active-Ambiguity correlation. It may be the case that early effects are acoustic and late effects are decision-related, but this hypothesis would need to be tested by, perhaps, orthogonally manipulating acoustic-phonetic properties and decision difficulty.

### Conclusions

In this study, exposure to isolated tokens of an acoustic continuum resulted in patterns of brain activity that reflected the subjective experience of categorical perception. Stimulus information persisted longer in active categorization trials than in passive listening trials, but, contrary to predictions, did not converge on discrete phoneme categories. Instead, in both tasks, a prolonged representation of the ambiguity vs. unambiguity of continuum stimuli emerged. Importantly, such a representation requires that subphonemic detail is not lost during the categorization process. Taken together, these neural decoding findings are consistent with parallel processes operating on speech input, yielding concurrent phonemic and subphonemic representations.

## ACKNOWLEDGMENTS

The authors thank the staff of the Athinoula A. Martinos Imaging Center at the McGovern Institute for Brain Research (MIT) for technical support. This work benefited from comments from two anonymous reviewers and helpful discussions with Jonathan Che, Yanke Song, Satrajit Ghosh, Tyler Perrachione, Stefanie Shattuck-Hufnagel, and Emily Myers and members of her lab.

## FUNDING INFORMATION

Research reported in this publication was supported by the Eunice Kennedy Shriver National Institute of Child Health and Human Development of the National Institutes of Health under award numbers F31HD100101 (to Sara D. Beach) and F32HD100064 (to Ola Ozernov-Palchik), and by MIT Class of 1976 Funds for Dyslexia Research (to John D. E. Gabrieli). Sara D. Beach also received support from the Friends of the McGovern Institute (MIT) and the Harvard Brain Science Initiative. The content is solely the responsibility of the authors and does not necessarily represent the official views of the NIH.

## AUTHOR CONTRIBUTIONS

**Sara D. Beach**: Conceptualization: Equal; Data curation: Equal; Formal analysis: Lead; Funding acquisition: Supporting; Investigation: Equal; Methodology: Equal; Project administration: Equal; Validation: Lead; Visualization: Lead; Writing–Original Draft: Lead; Writing–Review & Editing: Equal. **Ola Ozernov-Palchik**: Conceptualization: Equal; Funding acquisition: Supporting; Investigation: Equal; Project administration: Equal; Writing–Review & Editing: Equal. **Sidney C. May**: Data curation: Equal; Investigation: Equal; Project administration: Equal; Writing–Review & Editing: Equal. **Tracy M. Centanni**: Conceptualization: Equal; Investigation: Supporting; Project administration: Equal; Writing–Review &Editing: Equal. **John D. E. Gabrieli**: Conceptualization: Equal; Funding acquisition: Lead; Resources: Equal; Supervision: Lead; Writing–Review & Editing: Equal. **Dimitrios Pantazis**: Methodology: Equal; Resources: Equal; Software: Lead; Validation: Supporting; Visualization: Supporting; Writing–Original Draft: Supporting; Writing–Review & Editing: Equal.

## TECHNICAL TERMS

Phonemes: Individual speech sounds: vowels and consonants. A phoneme is the smallest unit of speech that distinguishes two words in a language, such as the /b/ in bin vs. the /d/ in din.
Magnetoencephalography (MEG): A functional brain-imaging technology that noninvasively records the magnetic fields generated by neurons’ electrical activity.
Neural decoding: Describes the attempt to determine what stimulus, event, or intent elicited a given pattern of brain activity.
Multivariate pattern analysis: A method of identifying patterns across many independent variables for the purpose of determining what information is contained in their combined fluctuations.
